# Supplementary material for: Use of the melting curve assay as a means for high-throughput quantification of Illumina sequencing libraries
Source: PeerJ. 2016 Aug 4;4:e2281. doi: 10.7717/peerj.2281 (PMC4991867; doi:10.7717/peerj.2281)

Supplementary Figure S1

(a)

PE1_adp_(+) oligo: 5’-A*C*A*C*T*T*TCCCTACACGACGCTCTTCCGATC*T-3’

PE2_adp_(-) oligo: 5’-/5Phos/GATCGGAAGAGCACACGTCTGAACT*C*C*A*G*T*C-3’

(b)

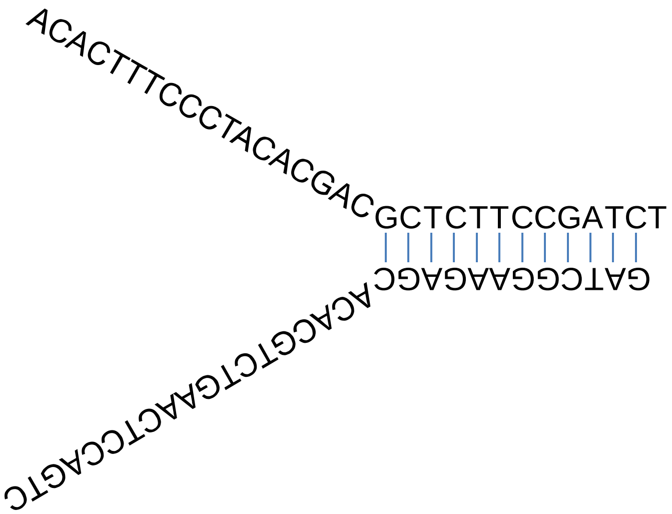

Supplement: Figure S1 — Sequence and structure of in-house Y-shaped adaptor. (A) The PE1_adp_(+) and PE2_adp_(-) oligonucleotides were ordered from Integrated DNA Technologies (IDT; IA, USA). Asterisk (*) between nucleotides denotes a phosphorothioate modification. (B) PE1_adp_(+) and PE2_adp_(-) oligonucleotides were annealed through incubation at 94°C for 1 minutes, and subsequent cool to 37°C for 30 mins. Annealed nucleotides are connected with a light blue line. [file peerj-04-2281-s001.pdf]
