# Supplementary material for: Use of the melting curve assay as a means for high-throughput quantification of Illumina sequencing libraries
Source: PeerJ. 2016 Aug 4;4:e2281. doi: 10.7717/peerj.2281 (PMC4991867; doi:10.7717/peerj.2281)

Supplementary Figure S2

(a)

Short DNA fragments

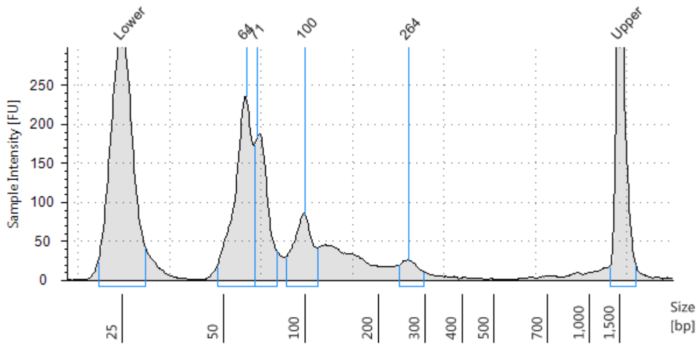

Single sequencing library

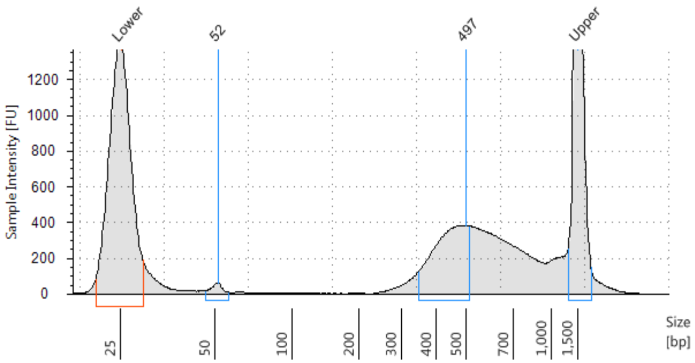

KAPA fragment

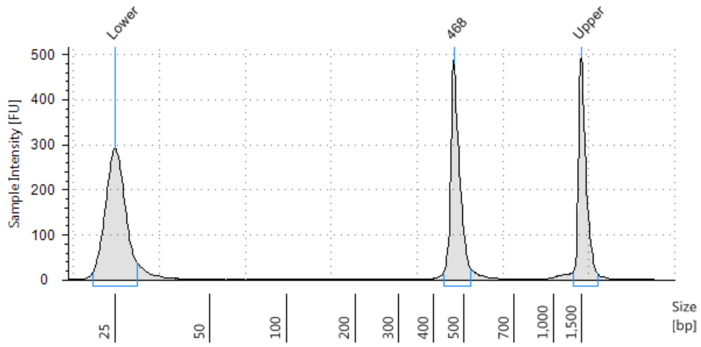

Supplementary Figure S2

(b)

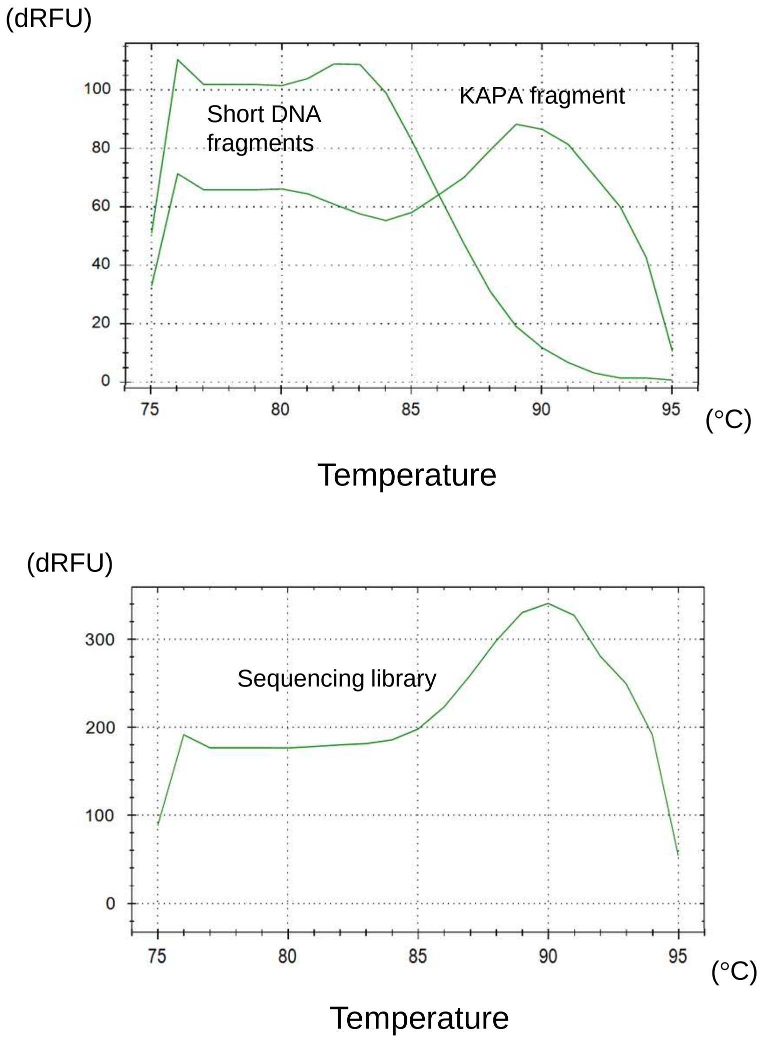

Supplementary Figure S2

(c)

Short DNA fragments

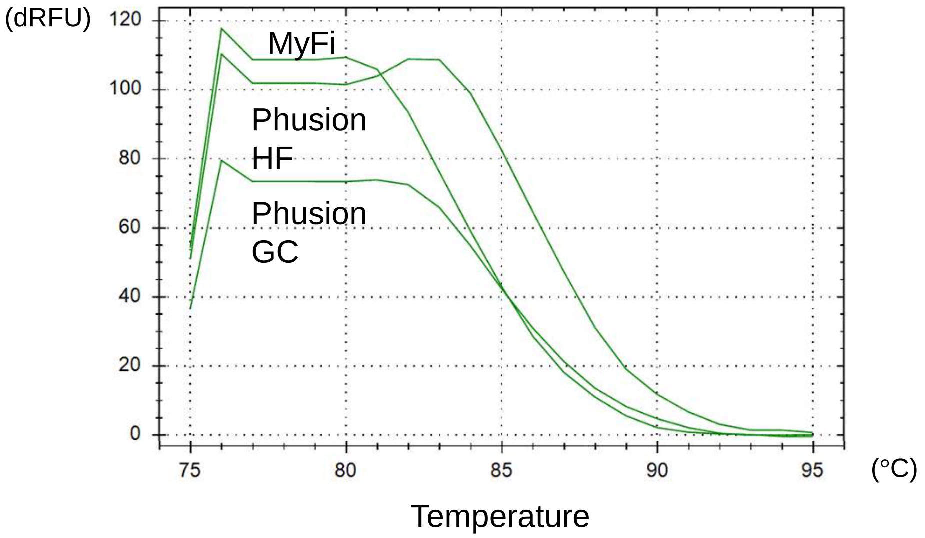

KAPA fragment

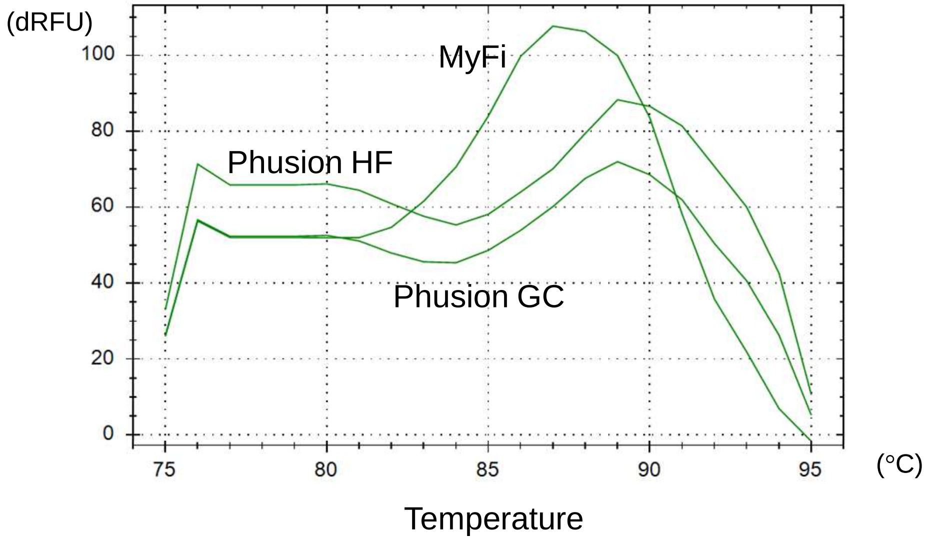

Supplement: Figure S2 — Results of the TapeStation and MC assay. (A) The TapeStation results for the short DNA fragments, KAPA fragment and a single sequencing library, showing size distribution of dsDNA. The High Sensitivity D1000 kit (Agilent) was used for the short DNA and KAPA fragments. The y = 0 and x = 0 axes show the fluorescence intensity and DNA fragment size, respectively. ‘Lower’ and ‘Upper’ indicate signal peaks of upper and lower DNA size markers, respectively. (B) The melting peak plots from the three samples obtained for temperatures between 75 and 95°C. (C) Effect of PCR buffer on MT of the short DNA and KAPA fragments. The MC assay was performed using three PCR buffers; the Phusion DNA polymerase HF and GC buffers and MyFiTM DNA polymerase buffer (Bioline, London, UK). [file peerj-04-2281-s002.pdf]
