# Supplementary figures and images for: Use of the melting curve assay as a means for high-throughput quantification of Illumina sequencing libraries
Source: PeerJ. 2016 Aug 4;4:e2281. doi: 10.7717/peerj.2281 (PMC4991867; doi:10.7717/peerj.2281)

## Supplementary Figure S3

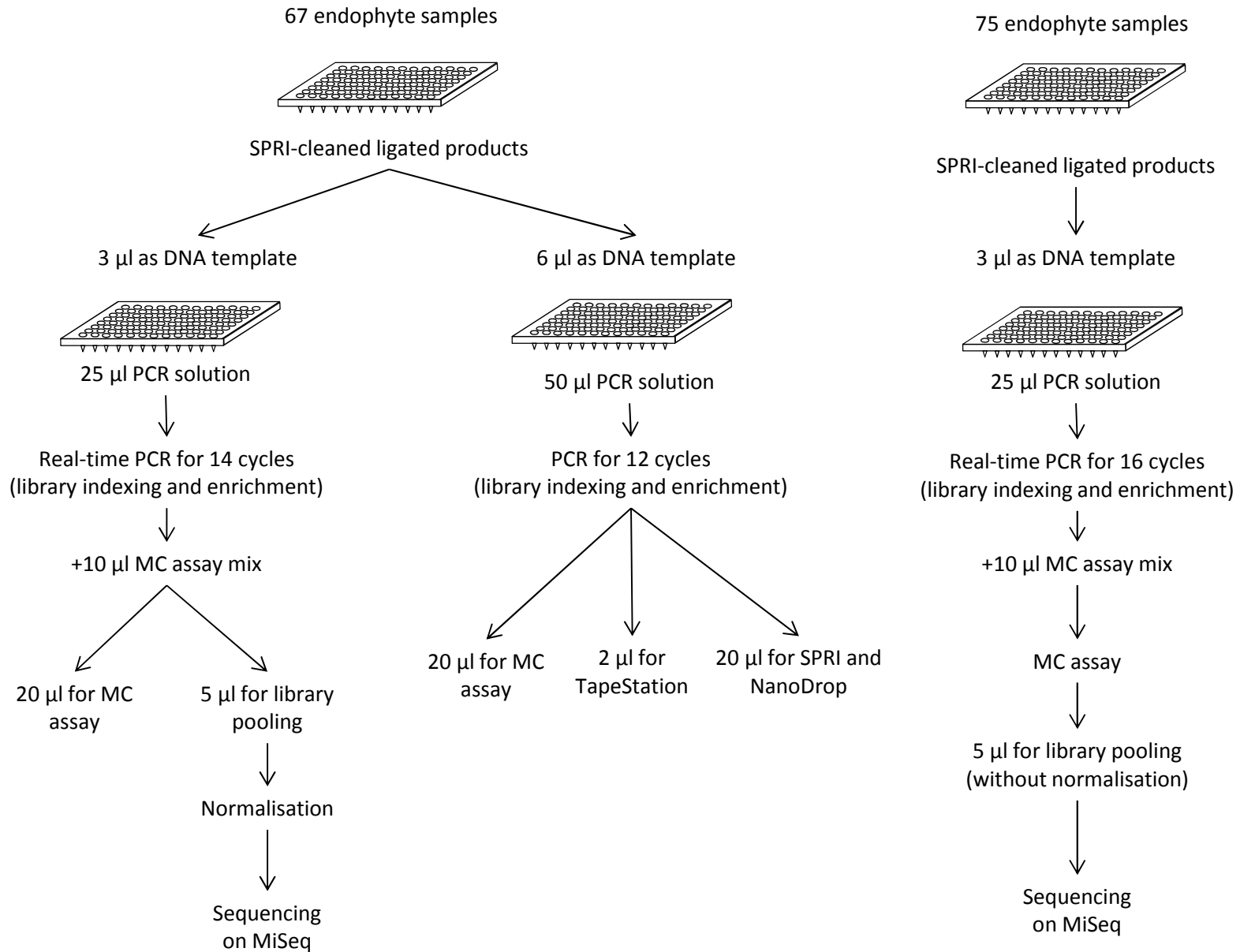

Supplement: Figure S3 — Schematic explanation for the library quantification experiments and library pooling procedure [file peerj-04-2281-s003.pdf]

## Supplementary Figure S4

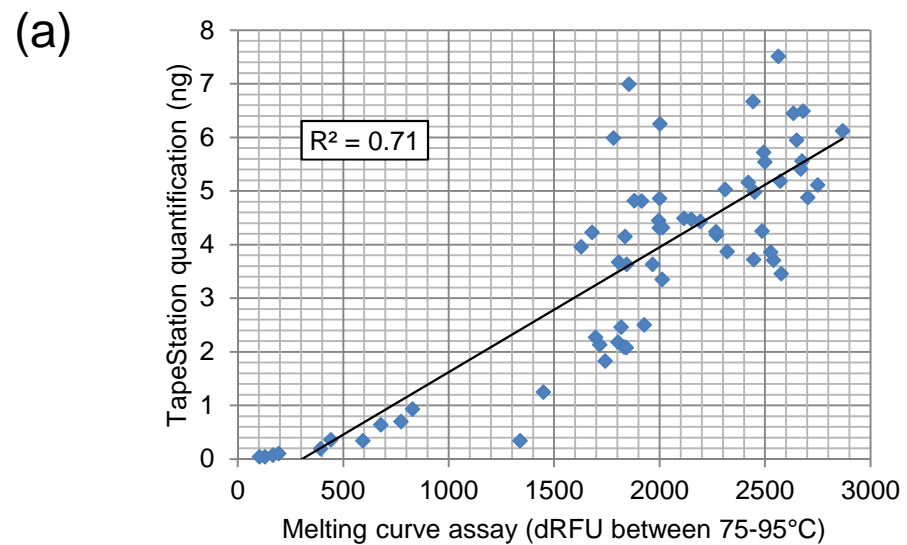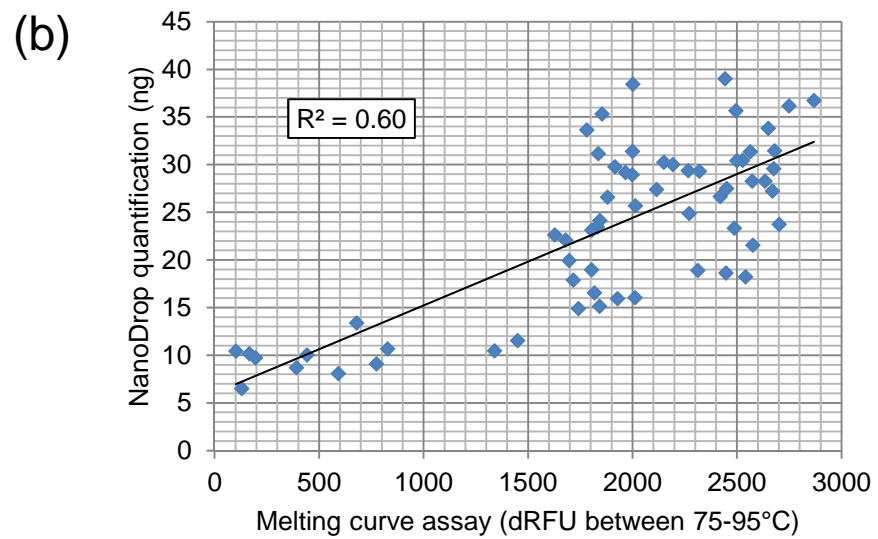

Supplement: Figure S4 — Correlation analysis between the sum of dRFU values between 75 and 95°C and quantification results from the TapeStation (A) and NanoDrop-based (B) methods [file peerj-04-2281-s004.pdf]
