# Supplementary material for: Use of the melting curve assay as a means for high-throughput quantification of Illumina sequencing libraries
Source: PeerJ. 2016 Aug 4;4:e2281. doi: 10.7717/peerj.2281 (PMC4991867; doi:10.7717/peerj.2281)
Supplement: Figure S5 — Coefficient of determination (R2) between the PRN from MiSeq sequencing and sum of dRFU values, depending on temperature ranges of the MC assay. The vertical and horizontal axes denote lower and upper range of the temperatures (°C) of the MC assay, respectively. The highest coefficient of determination (R2 = 0.823), which is from the PRN and dRFU values between 85 and 96°C, is marked with an asterisk. [file peerj-04-2281-s005.pdf]

Supplementary Figure S5

(°C)

	75	76	77	78	79	80	81	82	83	84	85	86	87	88	89	90	91	92	93	94	95
75	0.109	0.109	0.109	0.109	0.109	0.109	0.109	0.111	0.115	0.125	0.146	0.183	0.238	0.308	0.386	0.458	0.517	0.558	0.590	0.609	0.613
76		0.109	0.109	0.109	0.109	0.109	0.109	0.111	0.115	0.126	0.148	0.187	0.244	0.317	0.397	0.471	0.530	0.571	0.602	0.622	0.625
77			0.109	0.109	0.109	0.109	0.109	0.111	0.116	0.128	0.153	0.196	0.259	0.339	0.424	0.500	0.559	0.601	0.631	0.650	0.653
78				0.109	0.109	0.109	0.109	0.111	0.117	0.131	0.159	0.206	0.276	0.362	0.452	0.530	0.589	0.630	0.659	0.677	0.680
79					0.109	0.109	0.109	0.111	0.119	0.135	0.166	0.220	0.297	0.391	0.485	0.563	0.622	0.660	0.688	0.705	0.708
80						0.109	0.109	0.112	0.121	0.139	0.176	0.237	0.324	0.425	0.522	0.600	0.656	0.692	0.717	0.732	0.735
81							0.109	0.113	0.124	0.147	0.190	0.261	0.358	0.466	0.565	0.640	0.691	0.724	0.746	0.760	0.762
82								0.117	0.132	0.159	0.211	0.295	0.405	0.518	0.614	0.683	0.727	0.755	0.774	0.785	0.787
83									0.146	0.182	0.248	0.348	0.468	0.582	0.668	0.725	0.760	0.782	0.796	0.805	0.807
84										0.222	0.307	0.424	0.548	0.649	0.718	0.760	0.786	0.801	0.811	0.818	0.819
85											0.397	0.523	0.633	0.710	0.756	0.783	0.799	0.810	0.817	0.822	0.823*
86												0.625	0.704	0.752	0.778	0.793	0.803	0.811	0.816	0.820	0.821
87													0.749	0.773	0.786	0.795	0.802	0.808	0.812	0.815	0.816
88														0.781	0.788	0.794	0.800	0.805	0.809	0.811	0.812
89															0.789	0.794	0.800	0.804	0.808	0.809	0.809
90																0.797	0.802	0.805	0.808	0.808	0.808
91																	0.805	0.808	0.808	0.807	0.804
92																		0.808	0.806	0.800	0.796
93																			0.802	0.789	0.778
94																				0.759	0.731
95																					0.556

(°C)
